# Intention of physicians to implement guidelines for screening and treatment of latent tuberculosis infection in HIV-infected patients in The Netherlands: a mixed-method design

**DOI:** 10.1186/s12889-016-3539-2

**Published:** 2016-09-01

**Authors:** Kirsten Evenblij, Annelies Verbon, Frank van Leth

**Affiliations:** 1 Amsterdam Institute for Global Health and Development, Trinity Building C, Pietersbergweg 17, 1105 BM Amsterdam, The Netherlands; 2 Department of Internal Medicine, Division of Infectious Diseases, Erasmus Medical Center, Rotterdam, The Netherlands; 3 Department of Global Health, Academic Medical Center, University of Amsterdam, Amsterdam, The Netherlands

**Keywords:** Tuberculosis, HIV, Guidelines, Screening, LTBI, Mixed-method, Guideline implementation

## Abstract

**Backgound:**

All newly diagnosed HIV-infected patients in the Netherlands should be screened for latent tuberculosis infection (LTBI) and offered preventive therapy if infected without evidence of active tuberculosis. This guideline, endorsed by the national professional body of HIV physicians is in line with international recommendations, and based on the increased risk of progression from LTBI to active tuberculosis in HIV-infected patients. The objective of the study is to assess the intention of HIV physicians to implement this national guideline.

**Methods:**

A mixed method design triangulating results from two surveys among all (*n* = 80) HIV physicians in The Netherlands and qualitative interviews among 11 Dutch HIV physicians performed in 2014.

**Results:**

The majority of physicians used a risk-stratification approach based on individual a priori risk of tuberculosis to identify HIV-infected patients for LTBI screening, rather than screening all new HIV-infected patients. The intended and actual provision of preventive treatment was low, due to expressed doubts on the accuracy of diagnostic tools for LTBI. Interviewees reported that the guidelines did not match their clinical experience and lacked evidence for the recommendations. Screening for and treatment of LTBI was approached at a patient-level only. None of the interviewees referred to potential public health implications of the guidelines.

**Conclusions:**

Intended implementation of the national HIV-TB guidelines in the Netherlands is poor, due to a disconnect between clinical practice and evidence-based recommendations in the guideline. There is an urgent need to reconcile the views of HIV-physicians, public health experts, and guideline committee members, regarding the best strategy to address HIV-TB co-infection in the Netherlands.

**Electronic supplementary material:**

The online version of this article (doi:10.1186/s12889-016-3539-2) contains supplementary material, which is available to authorized users.

## Background

In an attempt to reduce the risk of active tuberculosis (TB), international guidelines stipulate screening HIV-infected individuals for latent TB infection (LTBI), and to offer preventive treatment if found infected and without evidence of active TB [1, 2]. The World Health Organization reinforces this strategy in its most recent guideline for tuberculosis control [3].

Although combination antiretroviral therapy (cART) has been shown to reduce the risk of developing TB in HIV-infected individuals, the risk of TB in HIV-infected individuals using cART remains higher compared to the risk in non HIV-infected individuals [4]. Preventive therapy in patients using cART has an independent and additional effect on the reduction of TB in HIV-infected patients [5], making LTBI screening and treating a worthwhile strategy in countries with a good access and uptake of ART.

There is no reference test for the diagnosis of LTBI. The currently available tests assess the presence of an interferon-gamma response after stimulation with *Mycobacterium tuberculosis* associated antigens in vivo (tuberculin skin test [TST]) or ex-vivo (Interferon-Gamma Release Assay [IGRA]). Testing by TST is hampered in immunosuppressed individuals due to possible anergy. IGRA test were thought to overcome this issue and therefore correlate better with the risk of progression to active TB. However, in a large European cohort study with more than 1700 patients with a wide variety of types of immunosuppression such superiority of IGRA over TST was not observed [6]. This equipoise makes that several international guidelines recommend either a two-step procedure (IGRA when TST-positive), or using both test simultaneously [7, 8].

In the Netherlands, HIV care is only provided in 27 designated HIV treatment centers (HTC). These centers are all in the public domain. HIV-physicians do provide other care within the field of internal medicine. All HIV physicians can be easily identified and approached, either through the designated HTC or through their professional body (Netherlands Association for HIV physicians [NVHB]. Treatment of patients with TB is in close collaboration with TB-experts in the Municipal Public Health Services throughout the country, regardless where the initial TB diagnosis is made. These services provide their support and expertise during the full duration of TB treatment.

In 2014, over 21,000 HIV-infected patients were registered, of whom 80 % were under clinical observation [9]. The Netherlands is considered to be a low incidence country with regards to tuberculosis, reporting an incidence of 1.9 per 100,000 population in 2014 [10]. The Commission for Practical Tuberculosis Control developed a national HIV-TB guideline which states that all newly diagnosed HIV-infected individuals should be screened for LTBI using both TST and IGRA, and be offered preventive therapy with nine months isoniazid (5 mg/kg/day and a maximum dose of 300 mg) if found infected without evidence of active TB (http://www.nvalt.nl/service/richtlijnen/richtlijnen/richtlijnen3/tuberculose---hiv) [11]. These guidelines were based on extensive systematic reviews and expert panel discussions, and endorsed by all relevant professional (para)-clinical bodies. The recommendation on the efficacy of and drug regimen for preventive treatment in HIV-infected patients was based on a systematic review by Akolo et al. [12]. Anecdotal evidence suggests that the recommendations regarding screening for and treatment of LTBI as defined in the guideline are not widely implemented.

The aim of this study is to evaluate the intentional and actual implementation of the Dutch HIV-TB guideline recommendations with respect to screening for and treatment of LTBI in newly diagnosed HIV-infected patients.

## Methods

We used an explanatory sequential mixed-methods design involving two phases [13]. First, a cross-sectional survey was conducted to i) explore the intention to screen for LTBI, ii) explore the intention to initiate preventive therapy, and iii) explore factors influencing intended guideline adherence. Second, in-depth interviews were held to thoroughly explore the survey findings and to get an understanding of physicians’ attitude and actual practice with regard to LTBI screening and treatment and the HIV-TB guideline. Data were collected between January and May 2014. A detailed description of the questionnaires and interview guide can be found in the Additional file 1.

### Quantitative study

**Fig. 1:**
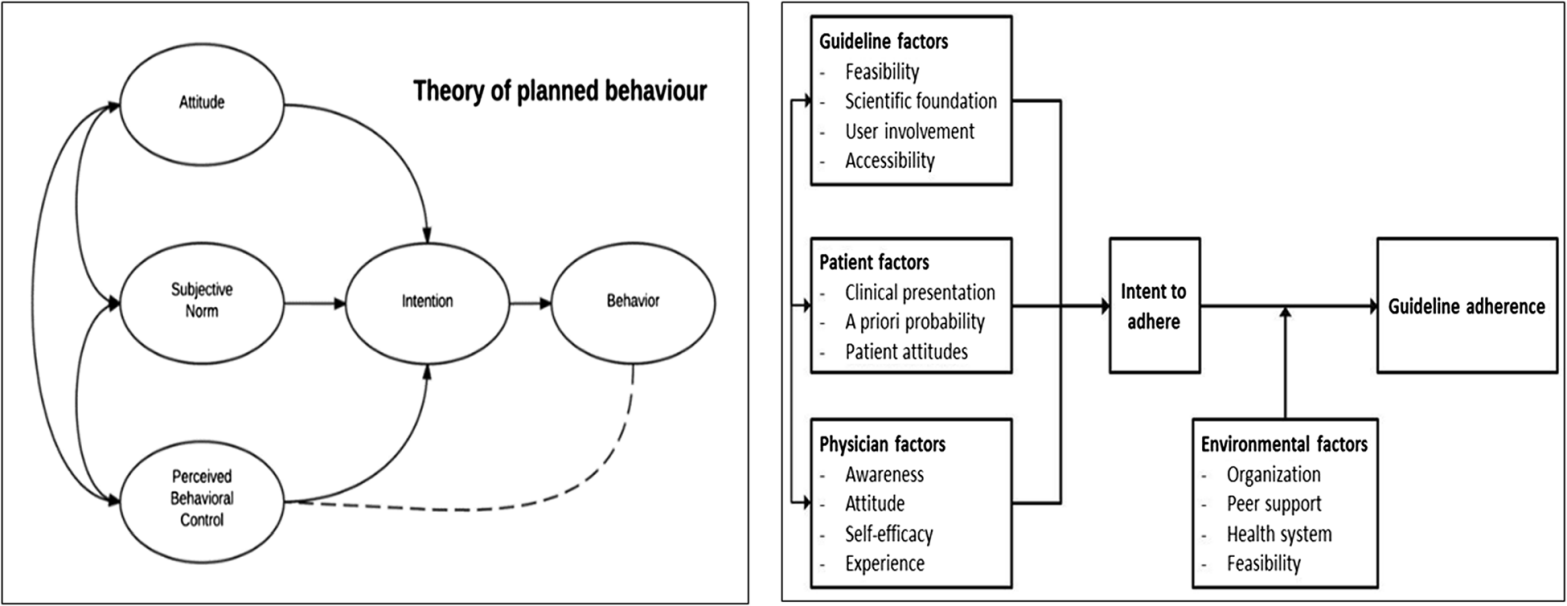
Theoretical framework used in study. *Left*: original description of Theopy of Planned Behavior (Ajzen et al [14]); *Right*: restructured framework to accommodate the assessment of a medical guideline

Quantitative data were collected through two cross-sectional surveys using questionnaires. The first questionnaire (”intention questionnaire”) assessed the intention to screen and treat for LTBI. It contained five hypothetical HIV-infected patient histories to assess the intention to screen for LTBI (screening cases [SC]). The histories were characteristic for specific risks for TB in HIV-infected individuals, and chosen to highlight the underlying evidence of the HIV-TB guidelines (Table 1). The intention to initiate preventive therapy given a specific screening result was assessed by formulating four treatment cases (TC) (Table 1). Responses to both screening and treatment scenarios was scored as yes/no/do not know. The questionnaire was self-administered and returned anonymously. The second questionnaire (“barrier questionnaire”) contained 22 statements on factors potentially influencing intended guideline adherence in line with the conceptual framework (Fig. 1). Statements could be answered with yes/neutral/no. Also this questionnaire was self-administered and could be returned (anonymously) by postal mail or email. The need for this information was identified very shortly after the initial results of the first questionnaire were received.

**Table 1:** Description of screening (SC) and treatment (TC) scenarios

Screening scenario	
SC I	Liberian man, 36 years, 3 months in The Netherlands, CD4 count 360/mm^3^
SC II	Liberian man, 36 years, 3 months in The Netherlands, CD4 count 90/mm^3^
SC III	Dutch woman, 42 years, intravenous drug use, CD4 count 450/mm^3^
SC IV	Dutch homosexual man, 26 years, CD4 count 20/mm^3^
SC V	Dutch heterosexual woman, 32 years, CD4 count 200/mm^3^
Treatment scenario	
TC I	TST positive (7 mm)
TC II	IGRA positive, TST negative (0 mm)
TC III	IGRA positive, TST positive (16 mm)
TC IV	IGRA negative, TST positive (16 mm)

The “intention questionnaire” was distributed, filled-in, and collected among the participants of the half-yearly meeting of the NVHB. The “barrier questionnaire” was sent to key persons in all HTC’s in the country with the request to distribute it among all HIV physicians in the facility, with several reminders by mail and phone to do so. The initial “intention questionnaire” was added to the distributed “barrier questionnaire” to give HIV physicians not present at the half-yearly NVHB meeting the opportunity to respond to the “intention questionnaire”. Following this approach, both questionnaires reached all 80 HIV-physicians in the country. The quantitative data from the questionnaires were analyzed with SPSS (version 22). Outcomes are reported as frequencies and percentages.

### Qualitative study

Qualitative data were collected through in-depth interviews with experienced HIV-physicians from HTCs to map factors influencing actual guideline adherence. The interview guide for these interviews was developed alongside the conduct of the quantitative surveys. Preliminary survey results were used as input for the interview guide which allowed us to get a more thorough understanding of the survey findings during the interviews. The interview guide was slightly adjusted after the analysis of the initial analysis of the surveys in order to obtain more specific information. In consecutive interviews, physicians were asked if they recognized specific issues identified through the surveys if they did not raise these issues themselves.

The in-depth and semi-structured interviews used a conceptual framework based on the Theory of Planned Behavior (TPB), which states that behavior is determined by a person’s behavioral intention and perceived behavioral control (or self-efficacy) [14]. We adapted the TPB model to fit the subject of medical guideline adherence. The original TPB concepts (attitude, subjective norm and perceived behavioral control) were reformulated to fit our research question, based on a literature study identifying factors influencing physicians’ guideline adherence in general and specifically for HIV-TB. This resulted in four main categories: i) guideline, ii) patient, iii) physician, and iv) environmental factors. By doing so, restructured categories relate better to daily clinical practice, and facilitate a targeted approach when addressing potential barriers. Furthermore, factors influencing the intention to adhere to the guideline (behavioral intention) were separated from factors influencing the actual use of the guideline (behavior). These latter factors are categorized under environmental factors. The general TPB framework remains the foundation of our conceptual framework: various sources of attitudes, subjective norms and measures of perceived behavioral control lead to an intent to behave and to potential behavior. Figure 1 depicts the original TBP model and our restructured model.

The interviews were conducted face-to-face, recorded, transcribed verbatim, and subjected to member check [15]. The study sample for the qualitative study was a convenience sample including experienced physicians, although care was taken to have a reliable spread with regards the geographic region, age, and work environment. The transcripts of the interviews were coded using thematic analysis by Braun and Clarke [16]. The coding scheme was developed using a semi-inductive approach, in which core themes were derived from the conceptual framework. The scheme was completed by themes that emerged during the analysis of the first two interviews. An iterative approach was used for the analysis of the qualitative data and terminated when saturation of the data was reached [17]. Themes were identified from the verbatim transcriptions and collected in an Excel worksheet, after which a manual appraisal of the themes and their frequency was conducted.

### Ethical approval

No ethical approval from the Netherlands Central Committee on Research Involving Human Subjects (CCMO) was needed. Dutch law states that “research which requires filling in a questionnaire just once generally does not fall under the scope of the Medical Research Involving Human Subjects Act (WMO)”, unless the “questions are detailed, burdensome, or intimate” (http://www.ccmo.nl/en/questionnaire-research). The Dutch law clearly states that therefore there is no need for an approval by a Medical Ethical Review Committee (METC). Mitigating circumstances that require METC approval despite not needing CCMO approval include the use of stored body material or issues arising under the Law on Medical Treatment Agreement (WGBO), which ensures an open and fair relationship between patient and physician. The interviewees not being patients and the absence of collecting body material, the mitigating reasons for METC approval are not present. With a written informed consent by each of the interviewees, a proper ethical standard under Dutch law for the study has been met.

## Results

### Quantitative study

Fifty-one of the 80 (64 %) “intention questionnaires”, and 24 (30 %) “barrier questionnaires“were returned. Eleven physicians, five females and six males, participated in an in-depth interview. They were from nine different hospitals from five cities (two small, three large). Their age varied between 40 and 61 years. The years of experience in the HIV-care varied between 10 and 29 years.

**Fig. 2:**
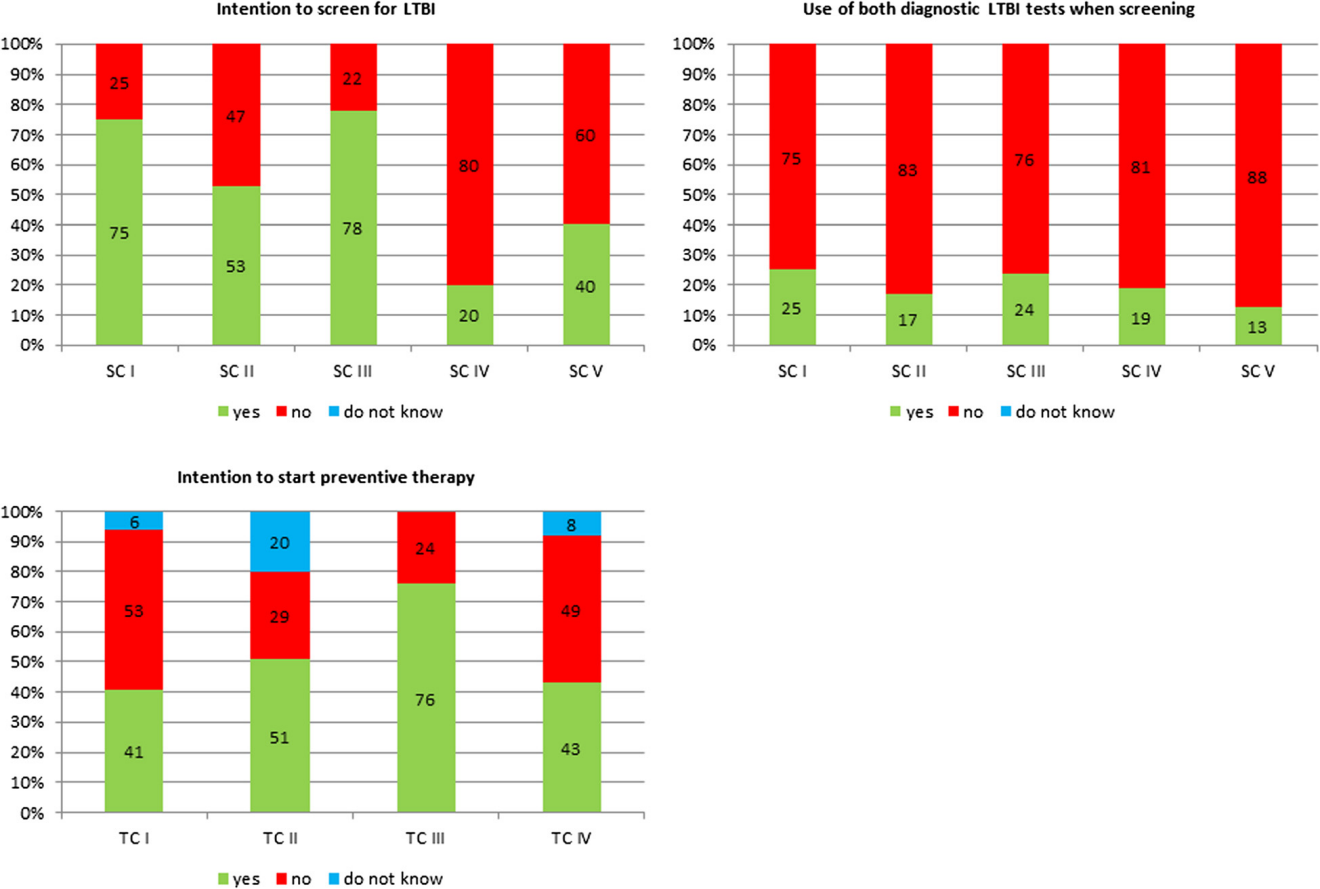
Intention to screen and start preventive therapy for hypothetical screening cases (SC) and treatment cases (TC). Definiton of SC and TC in Table 1. Upper left: intention to screen for LTBI, *Upper right*: intention to use both TST and IGRA test to assess LTBI, *Lower left*: intention to start preventive treatment if LTBI test positive without evidence of active TB

HIV-physicians intended to screen more frequently for LTBI in scenarios representing HIV-infected patients from Liberia, than Dutch HIV-positive patients, regardless of CD4 cell count (SC I: 38/51, 75 %, and SC II: 27/51, 53 %, versus SC IV: 15/51, 29 %, and SC V: 23/51, 40 %). The only exception is the frequent intention to screen when the Dutch HIV-infected patient is an intravenous drug user (SC III: 40/51, 78 %) (Fig. 2 Upper left). Although low CD4 count is a risk for progression from LTBI to active TB, respondents from both countries were less likely to be screened for LTBI in this setting (SC II: 27/51, 53 %, SC IV 15/51, 20 %), compared to those with high CD4 counts (SC I: 38/51, 75 %, and SC III: 40/51, 78 % and SC V: 23/51, 40 %, respectively). Of the 51 respondents, 12 (24 %) intended to screen for LTBI in all SCs as the guideline stipulates. Of the HIV-physicians who intended to screen for LTBI, less than 25 % would use both screening tests, as stipulated in the guideline, in any of the SCs (Fig. 2 Upper right).

HIV-physicians were less inclined to initiate preventive treatment in the treatment scenarios with an absent IGRA (TC I: 21/51, 41 %) or a negative IGRA (TC IV: 22/51, 43 %). When both IGRA and TST were positive (TC III), the majority (39/51, 76 %) of the HIV-physicians intended to initiate preventive treatment (Fig. 2 Lower left). One in five of the HIV physicians would not initiate preventive treatment in any of the treatment scenarios.

**Fig. 3:**
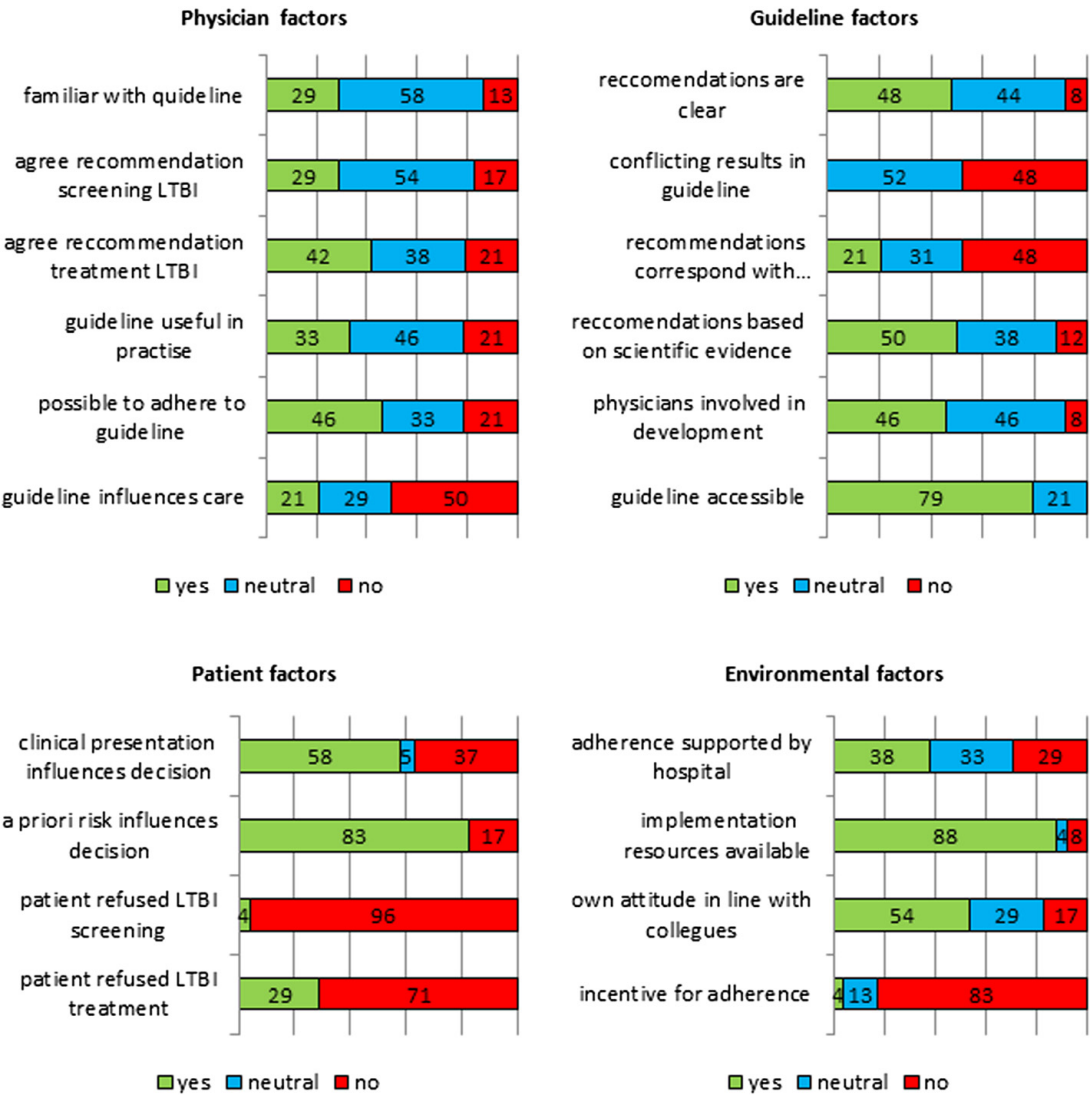
Barriers and facilitators for intended guideline adherence by factors indentified in theoretical framework. Figures are percentages. *Upper left*: physician factors; *Upper right*: guideline factors; *Lower left*: patient factors; *Lower right*: environmental factors

Responses to the statements relating to barriers and facilitators for guideline implementation are reported in Fig. 3.

#### Physician factors

The respondents answered in general neutrally on questions about their attitude towards the recommendations. In contrast, 12 of the 24 (50 %) respondents gave an outspoken negative answer on the question whether the guideline influenced their patient care (self-efficacy).

#### Guideline factors

Although almost none of the respondents considered the recommendations to be unclear, inaccessible, conflicting or insufficiently based on scientific evidence, only 21 % (5/24) of the physicians reported the recommendations to correspond to the daily practice. Adequate involvement with the formulation of the guideline (in person or through the professional body) was reported by 46 % (11/24) of the respondents, while 8 % (2/24) denied any involvement at all.

#### Patient factors

The a priori risk for LTBI was reported to drive the decisions regarding intended screening and treatment for LTBI, according to 83 % (20/24) of the respondents, while the clinical presentation of patients was a decisive factor for 58 % (14/24) of the respondents. Although the respondents reported that patients almost never refuse screening for LTBI, they do sometimes refuse preventive treatment.

#### Environmental factors

The large majority of the respondents (21/24, 88 %) reported that resources for the execution of the recommendations are available and accessible (time, space, staff, equipment). There are no incentives for adherence to the guideline. Four of the 24 (17 %) respondents reported to have a view on screening and treatment of LTBI that differed from their direct colleagues, indicating a large coherence within an HIV treatment facility.

### Qualitative study

The interviews showed that most respondents were aware of the HIV-TB guideline, but were not familiar with the recommendation regarding LTBI screening in all newly diagnosed HIV-infected patients. All interviewees showed a much more negative attitude towards the guideline than reported by the questionnaires, and considered the recommendations to be aimless and overdone, and none of the respondents adhered to it. Actual non-adherence was driven by the conviction that screening would not lead to extra health benefit, given (i) the perceived low a priori risk for LTBI in the Dutch population as the majority of HIV-infected individuals being Dutch gay men, (ii) the preventive effect of cART on the risk of TB, and (iii) the absence of actual TB diagnoses in their own practice in HIV-infected patients who are under regular follow-up. Other reasons for actual non-adherence included the fear of over-treating patients, and the unreliability of the screening tests. The value of doing both screening tests was contested by half of the non-screening physicians, who would prefer IGRA above TST because of its practical advantages and assumed better test characteristics.

Physicians were reluctant to initiate preventive therapy in HIV-infected patients on cART because of (i) the fear of potential side-effects and drug-interactions; (ii) the risk of over-treatment due the high amount of false positive test results, (iii) the perceived low risk that LTBI would progress to active TB, and (iv) the fear of poor patient compliance and the short duration of protection which could both increase the risk of drug-resistance.

### Data triangulation

In general, the opinions expressed during the interviews were more negative towards the underlying evidence for the guideline, and its relationship with clinical practice than elucidated from the questionnaires. Especially the latter issue was reiterated several times during each interview, and proved to be the driving force in institutional discussions on the use of the guideline in daily clinical work. The interviews identified in more detail the reasons behind the preference of the IGRA test over the TST in assessing LTBI, pointing strongly towards the commonly known logistical limitations of the TST (patients need to return to have the test assessed). Triangulation of the data elucidated the, commonly known, arguments against the use of preventive treatment in HIV-infected patients (side-effects, drug-interactions, and patients’ adherence). The interviewees corroborated the views on barriers to guideline implementation as seen in the questionnaire. They reported to be confused by multiple (inter)national guidelines, which are often contradictory and not up-to-date. For example, the latest version (2013) of the HIV-TB guideline, including the recommendations on preventive therapy, had not yet been posted on the website of the NVHB at the time of the study (2014). The majority of the respondents had missed a joint discussion on the guidelines within their professional body, potentially leading to a lack of consensus about screening and treatment policies for LTBI.

## Discussion

This study showed that Dutch HIV physicians have a low intention to implement the HIV-TB guideline recommendations on screening for and treatment of LTBI. Physician’s risk assessment on LTBI and progression to active TB differs substantially from the risk assessment used to underline the national (and international) guidelines. This mismatch does not imply poor clinical practice, as the study was not designed to be a clinical audit, but solely an assessment of implementation.

A similar assessment of LTBI screening and treatment practices has been reported by Wyndham-Thomas et al. [18]. The study was carried out in Belgium, which is, just as the Netherlands, a low-incidence country for tuberculosis, and showed similar findings. The authors reported marked inconsistencies in screening and treatment approaches among HIV-physicians. Overall, just 20 % of the physicians would screen for LTBI, and 14 % would use both diagnostic test. Interestingly is that the authors attribute these inconsistencies to the absence of up-to-date guidelines. Our study shows that even with up-to-date guidelines that are endorsed by the professional body of HIV physicians, intended and actual adherence is poor.

Data from the Swiss HIV Cohort Study showed that physicians were more likely to screen HIV-infected patients with high a priori risks and high CD4 counts [19]. This cohort uses routine data and is therefore a useful source to explore routine clinical behavior of HIV physicians, even if studies are not directly designed to do so. Our finding that even amongst high risk patients, respondents were more frequently intended to screen for LTBI in patients with high rather than low CD4 counts can be explained by assuming that physicians are aware of test limitations [12, 20, 21]. Despite differences in test characteristics, it remains unclear how useful either test (TST or IGRA) is in identifying those patients with immunosuppression at highest risk for progression to active TB. The aforementioned study by Sester et al. showed that neither TST, nor any of the IGRA tests had this ability. Three out of 11 TB patients identified within a period of two years after the initial test had a negative test result for TST and two IGRAs [6]. A post-hoc analysis from the same study showed that actual HIV viral load in plasma at the time of LTBI test performed better in separating those with a higher risk of progression from those with a low risk [22]. The results from our questionnaire indicated that, if anything, respondents had most faith in the results of an IGRA test in their decision to start preventive therapy or not.

The finding of poor intended guideline implementation, and the actual non-adherence the guideline as reported by the interviewees in our study agrees with previous studies on adherence to clinical guidelines in general, and guidelines specific for LTBI [23, 24]. Physician related barriers to intended and actual guideline adherence included a lack of awareness of, and agreement with the relevant recommendations [23, 25], lack of self-efficacy [23], and absence of direct experience with TB in their own practice [24]. The main guideline related barriers were the perceived poor correspondence of the guideline to daily practice, and the assumed low quality and quantity of the scientific evidence presented in the guideline. Such barriers affect heavily successful implementation [26–28]. The strong negative attitude towards the guidelines expressed during the interviews was only partly validated by the results of the questionnaires, which makes interpretation difficult. Although 8 % of the survey respondents felt insufficiently involved in the guideline development, almost all interviewees complained about a lack of end-user involvement. Involvement of the end-user benefits guideline adherence by an increased sense of ownership [26]. Representatives from the professional group of HIV physicians were part of the guideline committee from the start, while the guidelines were presented, and adopted in one of the half-yearly professional meetings.

The difference in intention to screen between HIV-infected patients from Liberia and the Netherands is most likely due to a perceived low a priori risk of LTBI in Dutch HIV-infected patients. It is reported as one of the most important patient-related barriers to guideline adherence, as seen in other European countries [29]. The respondents thereby fail to acknowledge that missing a chance to prevent a case of TB has noticeable consequences at the population level, given ongoing transmission [30]. Almost one in three patients with pulmonary TB in The Netherlands in 2014 had a patient delay (onset cough to first visit care provider), while more than one in four had a doctor’s delay (from first consultation to start treatment) of more than 8 weeks [10].

The main strength of the study was a design enabling data triangulation. The interviews confirmed the respondents’ intended non-adherence to the guideline, and enriched the data by giving insight in the actual attitude towards the recommendations and reasons for non-adherence. Adding the qualitative data to the results of the questionnaires identified a deep resentment of HIV physicians towards the guideline. Something that could not be distilled from the questionnaires alone. This has an impact on actions to be taken. Just discussing the need for implementation is bound to fail when the resentment toward the guideline is that deep. The triangulation of the data makes that a renewed and rigorous discussion on the need, content, and implementation of any HIV-TB guideline in The Netherlands is needed. This discussion has started.

Solely relying on the results from a questionnaire in a complex assessment like intended and actual clinical management runs the risk of having data that are difficult to interpret. Such situation occurred in the study from Belgium referenced earlier, in which almost one third of the questionnaires had inconsistent answers resulting from not following questionnaire guidance.

Although in practice domains used in the revised theoretical are likely not to be that clearly demarcated, the framework provided a structure and enabled us in both the quantitative and the qualitative study to disentangle the different domains of potential factors influencing intended and actual guideline adherence.

The main limitation of the study is the sub-optimal sampling method. For the quantitative study, non-response was solely due to not returning the questionnaires. Due to anonymity, we could not assess potential bias in physicians responding and those not. The results obtained from the questionnaire fit the anecdotal evidence towards testing and treating of LTBI in the professional group, and covers practices in 19 of the 27 HIV-treatment facilities in The Netherlands. Discussing the findings with the HIV physicians strengthens our believe that we were able to capture the prevailing opinions with respect to intended screening and treatment behavior. Although this can not serve as evidence for representativeness, it seems very unlikely that a different picture would have emerged when a larger number of physicians would have participated. The qualitative part of the study uses a well-validated approach in which key-informants are interviewed until responses are saturated. The choice to use experienced HIV-physicians was taken to allow for opinions known to have a strong impact on clinical practice of younger colleagues. Screening and treatment strategies are organized at an institutional level. Using key informants gave us the opportunity to discuss these approaches in detail. Despite these sources of potential selection bias, we feel that the results accurately highlight the level of intended and actual guideline implementation and the perceived barriers and facilitators to implementation.

## Conclusions

There is an urgent need for an in-depth discussion on the perceived mismatch between clinical risk-assessment by Dutch HIV-physicians, and the risk assessment based on the scientific evidence used for the Dutch HIV-TB guideline. Furthermore, the need, content and eventual implementation strategy needs to be reconsidered by a discussion between the guideline committee and the professional body of HIV physicians.

## Additional file

Additional file 1: Questionnaires and interview guide. (DOCX 21 kb)

## Abbreviations

cART: Combination antiretroviral therapy
HIV: Human immunodeficiency virus
HTC: HIV Treatment center
IGRA: Interferon gamma release assay
LTBI: Latent tuberculosis infection
NVHB: Netherlands Association for HIV physicians
TB: Tuberculosis
TST: Tuberculin skin test
