# Supplementary material for: Intention of physicians to implement guidelines for screening and treatment of latent tuberculosis infection in HIV-infected patients in The Netherlands: a mixed-method design
Source: BMC Public Health. 2016 Sep 1;16(1):915. doi: 10.1186/s12889-016-3539-2 (PMC5009683; doi:10.1186/s12889-016-3539-2)
Supplement: Additional file 1: — Questionnaires and interview guide. (DOCX 21 kb) [file 12889_2016_3539_MOESM1_ESM.docx]

# Supplemental file: Questionnaires and interview guide

**Questionnaire 1: Screening scenarios**

1. Which test, TST or IGRA, do you prefer for diagnoses of LTBI at the following patients who have nog yet been treated for an HIV infection?
2. 36-year old man, coming from Liberia, since 3 months in the Netherlands. Newly diagnosed with HIV, CD4 count of 360x10^6^/L lymphocytes
3. IGRA
4. TST
5. Both
6. None
7. 36-year old man, coming from Liberia, since 3 months in the Netherlands. Newly diagnosed with HIV, CD4 count of 90x10^6^/L lymphocytes
8. IGRA
9. TST
10. Both
11. None
12. 42-year old Dutch women, intravenous drug user. Newly diagnosed with HIV, CD4 count of 450x10^6^/L lymphocytes
13. IGRA
14. TST
15. Both
16. None
17. 26-year old Dutch MSM. Newly diagnosed with HIV, CD4 count of 20x10^6^/L lymphocytes
18. IGRA
19. TST
20. Both
21. None
22. 32-year old Dutch woman, heterosexual. Newly diagnosed with HIV, CD4 count of 200x10^6^/L lymphocytes
23. IGRA
24. TST
25. Both
26. None

**Questionnaire 1: Treatment scenarios**

Will you start, after exclusion of active TB, with preventative therapy of LTBI at a 36 year old man, coming from Liberia, who is since 3 months in the Netherlands with a newly diagnosed HIV infection with a CD4 count of 360x10^6^/L lymphocytes and

1. A TST of 7 mm
2. Yes
3. No
4. Do not know
5. A positive IGRA and negative TST (0 mm)
6. Yes
7. No
8. Do not know
9. A positive IGRA en positive TST (16 mm)
10. Yes
11. No
12. Do not know
13. A negative IGRA and positive TST (16 mm)
14. Yes
15. No
16. Do not know

**Questionnaire 2: Barriers and facilitators for the implementation of the Dutch HIV/TB guideline**

| Physicians factors | | Awareness | 1. Did you know of the existence of the Dutch HIV/TB guideline? | | yes / neutral / no |
| --- | --- | --- | --- | --- | --- |
|  |  |  | 1. Do you consult the Dutch HIV/TB guideline? | | yes / sometimes / never |
|  |  |  | 1. Are you familiar with the recommendations in the guideline? | | yes / partial / no |
|  |  | Attitude | 1. Do you agree with the guideline recommendations regarding screening for LTBI? | | yes / neutral / no |
|  |  |  | 1. Do you agree with the guideline recommendations regarding initiation of preventive therapy for LTBI? | | yes / neutral / no |
|  |  |  | 1. Do you think the guideline is useful in practice? | | yes / neutral / no |
|  |  | Self-efficacy | 1. Do you think it is possible to adhere to the guideline recommendations in practice? | | yes / neutral / no |
|  |  |  | 1. Do you think the guideline recommendations influence your patient care? | | yes / neutral / no |
| Guideline factors | | Feasibility | 1. Do you think the guideline recommendations are clear? | | yes / neutral / no |
|  |  |  | 1. Do you think the guideline reports conflicting results? | | yes / neutral / no |
|  |  |  | 1. Do you think the guideline recommendations correspond with the existing patient care? | | yes / neutral / no |
|  |  | Scientific basis | 1. Do you think the guideline recommendations are sufficiently based on scientific evidence? | | yes / neutral / no |
|  |  | Involvement end-user | 1. Do you think that you or the association of HIV practitioners was sufficiently involved in the development of the Dutch HIV/TB guideline? | | yes / neutral / no |
|  |  | Accessibility | 1. Do you think the HIV/TB guideline is accessible in your hospital? | | yes / neutral / no |
| Patient factors | | Clinical presentation | 1. Does the clinical presentation of a patient influence your decision to screen for and initiate treatment of latent tuberculosis? | | yes / neutral / no |
|  |  | A priori probability | 1. Does the a priori probability for LTBI of a patient (e.g. patient born in tuberculosis endemic country) influence your decision to screen for and initiate treatment of latent tuberculosis? | | yes / neutral / no |
|  |  | Patient attitudes | 1. Has it occurred that a patient refused screening for LTBI? | | yes / neutral / no |
|  |  |  | 1. Has it occurred that a patient refused preventive therapy for LTBI? | | yes / neutral / no |
| Environmental factors | Organizational | | 1. Do you think adherence to the guideline is supported in your organization? | yes / neutral / no | |
|  |  |  | 1. Do you think the resources are available to execute the guideline recommendations? |  | |
|  | Support of peers | | 1. Do you think your attitude towards the Dutch HIV/TB guideline is congruent with the attitude of your colleagues? | yes / neutral / no | |
|  | Health care system | | 1. Are there incentives (legally/financially) to adhere to the guideline? | yes / neutral / no | |
| Demographics | | | 1. What is your sex | Man / woman | |
|  |  |  | 1. What is your age? | …… years | |
|  |  |  | 1. How many years have you been practicing in HIV care? | …… years | |
| Patient brochure | | | 1. Do you think patients would like to receive a brochure about HIV/TB co-infection? | Yes / neutral / no | |

**Interview guide**

*Grand tour*

1. Can you please explain what usually happens when a new HIV patient visits the hospital with regard to LTBI/TB?
   1. Standard procedures
   2. Risk groups
2. What do you think about the treat of LTBI in HIV-positive patients

*Guideline implementation*

1. What do you think about the recommendations in the HIV/TB guideline regarding
   1. Screening for LTBI in all new HIV patients
   2. Screening with TST and IGRA
   3. Initiating IPT 9 months when IGRA and/or TST is positive
2. What are factors that influence your decision to follow the recommendations in the HIV/TB guideline?
   1. Physician factors
   2. Guideline factors
   3. Patient factors
   4. Environmental factors
3. What do you feel are advantages/disadvantages of guidelines?
4. To what extent do you feel the guideline is implemented in this HIV care center?
   1. Access
   2. Materials available to execute recommendations
   3. Use by colleagues
5. If you were asked to adapt the HIV/TB guideline regarding screening and initiating treatment for LTBI, what would you like to change

*End*

I do not have any questions anymore. Would you like to add something or ask something before ending this interview? Can I ask you what age you are and how long you have been working as an HIV physician? Thank you for participating in this research. I will send you a summary of this interview. If you have any questions please do not hesitate to contact me. Thank you again for your cooperation.
